# Phytochemical Profiling and Safety Assessment of *Maerua crassifolia* Forssk. Aerial Parts Infusion With Pharmacological Validation of Its Analgesic and Anti‐Inflammatory Activities

**DOI:** 10.1002/open.70326

**Published:** 2026-10-26

**Authors:** Leila Benazzi, Saida Ouafi, Redouane Semaoui, Faiza Baali, Jordi Eras, Ramon Canela‐Garayoa, Nadjette Djemouai

**Affiliations:** ^1^ Laboratory of Research on Arid Zones (LRZA) Faculty of Biological Sciences University of Science and Technology Houari Boumediene (USTHB) Algiers Algeria; ^2^ Department of Biology Faculty of Natural and Life Sciences and Earth Sciences University of Ghardaia Ghardaïa Algeria; ^3^ Department of Chemistry DBA center University of Lleida Lleida Spain; ^4^ Scientific Technical Services TCEM University of Lleida Lleida Spain; ^5^ Laboratory of Microbial Systems Biology (LBSM) Higher National School Cheikh Mohamed El Bachir El Ibrahimi Kouba, Algiers Algeria

**Keywords:** chemical composition, inflammation, *Maerua crassifolia* Forssk., pain relief, toxicity

## Abstract

*Maerua crassifolia* Forssk. is used in Algerian traditional medicine to relieve pain and manage inflammation. This study aimed to explore the composition, safety, in vitro antioxidant activity and in vivo analgesic, and anti‐inflammatory effects of the infusion of *M. crassifolia* aerial parts. The infusion yielded 11.93 ± 1.08% (w/w) and exhibited high total phenolic (620.51 ± 2.54 mg GAE/100 g dry weight (d.w.)) and flavonoid (87.11 ± 0.76 mg QE/100 g d.w.) contents. Ultra‐performance liquid chromatography‐photodiode array detection‐tandem mass spectrometry (UPLC‐PDA‐MS/MS) identified 19 phenolic compounds. The infusion showed strong antioxidant activity in the four assays tested. Acute oral toxicity testing showed a favorable safety profile (LD_50_ > 5000 mg/kg body weight (b.w.)). The results showed that the infusion exerted dose‐dependent analgesic activity, with the highest inhibition of abdominal writhing (74.02 ± 2.17%) and paw‐licking time (up to 77.78%) observed at 1000 mg/kg b.w., comparable to paracetamol, the standard analgesic drug. In the anti‐inflammatory test, the infusion produced dose‐dependent activity, with 44.42% ± 2.30% and 56.22% ± 1.52% inhibition at 500 and 1000 mg/kg b.w., respectively, comparable to the standard anti‐inflammatory drug sodium diclofenac. These findings position *M. crassifolia* as a promising candidate for the management of pain and inflammation.

## Graphical abstract

Rich in phenolic compounds, *Maerua crassifolia* Forssk. aerial parts infusion combines strong antioxidant capacity with a favorable acute safety profile. It produces dose‐dependent analgesic and anti‐inflammatory effects in vivo, approaching reference‐drug activity. These findings highlight this desert plant as a promising natural source of pain‐ and inflammation‐modulating compounds.

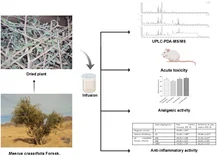

## 1 Introduction

Medicinal plants have profoundly shaped human health across civilizations, serving as the cornerstone of traditional healing systems long before modern pharmacology emerged. Alkaloids, terpenoids, flavonoids, and phenolics are among the bioactive compounds that provide a range of therapeutic advantages, like anti‐inflammatory and analgesic effects [1, 2]. Estimates from the World Health Organization (WHO) indicate that 80% of the global population, especially in low‐ and middle‐income countries, get their primary medical care from plant‐based therapies [3]. Beyond their role in traditional medicine, natural ingredients have played crucial roles in the identification and advancement of modern pharmaceuticals and continue to be a major source of novel therapeutic agents. Several classes of medicines, including antihypertensive, anti‐infective, antimigraine, and other drugs, have been derived from or inspired by natural products [4, 5]. These natural products have fueled drug discovery, with approximately 25% of modern pharmaceuticals derived directly from plants, with landmark examples like artemisinin (from the sweet wormwood plant, used in the treatment of malaria) [1, 6]. In addition to direct extraction, plant‐derived semi‐synthetic analogs and pharmacophores are pivotal in fostering innovation, generating over $100 billion annually in the global herbal medicine market, and inspiring targeted therapies for diabetes, cardiovascular disease, neurodegeneration, and other conditions [7].

Phenolic chemicals are among the most common and structurally varied families of secondary metabolic products in the kingdom of plants [8, 9]. Ubiquitously distributed across fruits, vegetables, leaves, bark, and roots, they serve critical ecological roles in safeguarding plants from infections, ultraviolet radiation, and herbivory, while conferring distinctive colors, flavors, and bitterness [8]. These substances’ numerous biological activities and technological uses have piqued scientists’ interest [10]. Chemically defined by aromatic ring structures, phenolics exhibit remarkable redox properties, facilitating their trapping of free radicals, chelating metals, and modulating key signaling pathways [11]. Their anti‐inflammatory properties arise from the inhibition of pro‐inflammatory cytokines and enzymes like cyclooxygenase‐2 (COX‐2), positioning phenolics as substitutes for synthetic nonsteroidal anti‐inflammatory pharmaceuticals [12]. Moreover, the analgesic attributes of these compounds stem from modulation of pain pathways, including TRPV1 receptor antagonism, opioid receptor activation, and inhibition of prostaglandin synthesis, with flavonoids like quercetin demonstrating efficacy in neuropathic and inflammatory pain models [13]. Contemporary investigative toxicology ensures safe translation of traditional plant remedies to clinical candidates through systematic toxicity profiling, which is essential given that high doses of certain phenolics, such as tannins and saponins, may induce gastrointestinal irritation, hepatotoxicity, or genotoxicity [14, 15].

Algeria’s diverse phytogeography, from Saharan oases and Atlas Mountains to Mediterranean coasts, harbors over 3,000 medicinal plant species integral to ancestral practices [16, 17]. Recent studies document hundreds of species used by Algerian populations, demonstrating promising therapeutic effects against various ailments [18, 19, 20, 21, 22, 23, 24]. Amongst these, *Maerua crassifolia* Forssk., a tree known in Arabic as “Atil” and in Tamahaq as “TEadjart” and belonging to the Capparaceae family, remains underexplored [25]. Extensively prevalent in arid areas, this species thrives under harsh environmental conditions, exhibiting remarkable tolerance to prolonged drought [26, 27]. In folk medicine, *M. crassifolia* decoctions are traditionally used to manage toothache, wounds, itching, skin eruptions, rheumatism, indigestion, diarrhea, abdominal pain, diabetes, fever, and malaria [28, 29]. These traditional uses, particularly those related to rheumatism, toothache, and painful conditions, suggest a potential role of the plant in the management of pain and inflammatory disorders. In addition, *M. crassifolia* has been reported as a food resource in arid and famine‐prone regions. Its leaves are rich in minerals such as calcium and iron, and amino acids such as phenylalanine and tyrosine, and in linoleic and alpha‐linolenic acids, which serve as a valuable food source in low‐income areas, further highlighting its ethnobotanical importance [30]. Despite its extensive traditional use, the biological properties of *M. crassi‍folia* Forssk. remain insufficiently investigated in Algeria, particularly regarding its analgesic and anti‐inflammatory potential. Therefore, further pharmacological investigation is warranted to provide scientific support for its traditional uses. Accordingly, this study explored the phytochemical composition, in vitro antioxidant properties, acute oral toxicity, and in vivo anti‐inflammatory and pain‐relieving attributes of *M. crassifolia* Forssk. aerial parts.

## 2 Materials and Methods

### 2.1 Ethics Statement

The experimental protocols were approved by the University of Sciences and Technology Houari Boumediene Animal Ethics Committee (approval ref. no. 162/2011/8) and are in accordance with the directives of the European Union regarding the handling of experimental animals (86/609/EEC).

### 2.2 Standards

Reference standards for compound identification were obtained from certified commercial suppliers. The analyzed panel included rutin, hyperoside, quercetin, phloretin, kaempferol, procyanidin A2, procyanidin B2, procyanidin C1, theaflavin digallate, caffeic, p‐coumaric, ellagic, salicylic, vanillic, and ferulic acids acquired from Chengdu Biopurify Phytochemicals Ltd. (Cymit Quimica, Barcelona, Spain). Chlorogenic and gallic acids were purchased from Acros Organics (Fisher Scientific SL, Madrid). Delphinidin‐3‐*O*‐rutinoside chloride, quercetin‐3‐*O*‐glucopyranoside, cyanidin‐3‐glucoside chloride, procyanidin B1, catechin, epicatechin, pelargonidin‐3‐rutinoside chloride, keracyanin chloride, luteolin, vitexin, eriodictyol, epicatechin gallate, luteolin‐7‐*O*‐glucoside, quercitrin, orientin, luteoloside, isorhamnetin glucoside, dicaffeoyl quinic acid, narirutin, vicenin II, and eriocitrin, were purchased from Extrasynthese (Lyon, France). In addition, tangeretin, hydroxytyrosol, daidzein, galangin, genistein, arbutin, fisetin, taxifolin, phloridzin, theaflavin, naringin, naringin dihydrochalcone, hesperidin, narcissoside, epigallocatechin gallate, and protocatechuic and syringic acids were purchased from Sigma–Aldrich Chemie (Steinheim, Germany). The stock solutions were diluted in methanol and kept at −80 °C. An aliquot of the stock solutions was diluted with the injection solvent, which was an aqueous solution of 0.1% acetic acid, to create working solutions.

### 2.3 Plant Material

Aerial plant materials comprising leaves and branches of *M. crassifolia* Forssk. were collected in January 2016 from Tissenaouene station in Tamanrasset Province, Algeria (latitude: 22°44′59″N, longitude: 5°27′07″E, and altitude: 1313 m) (Figure 1). The plant was authenticated by the Higher National Agronomic School (ENSA, Algiers, Algeria), and a voucher specimen (C 10002562) is found at the Herbarium of the Museum Botanicum Hauniense, University of Copenhagen, Denmark. Aerial parts were desiccated in the shade at ambient temperature, ground, and kept at room temperature pending use.

**FIGURE 1:**
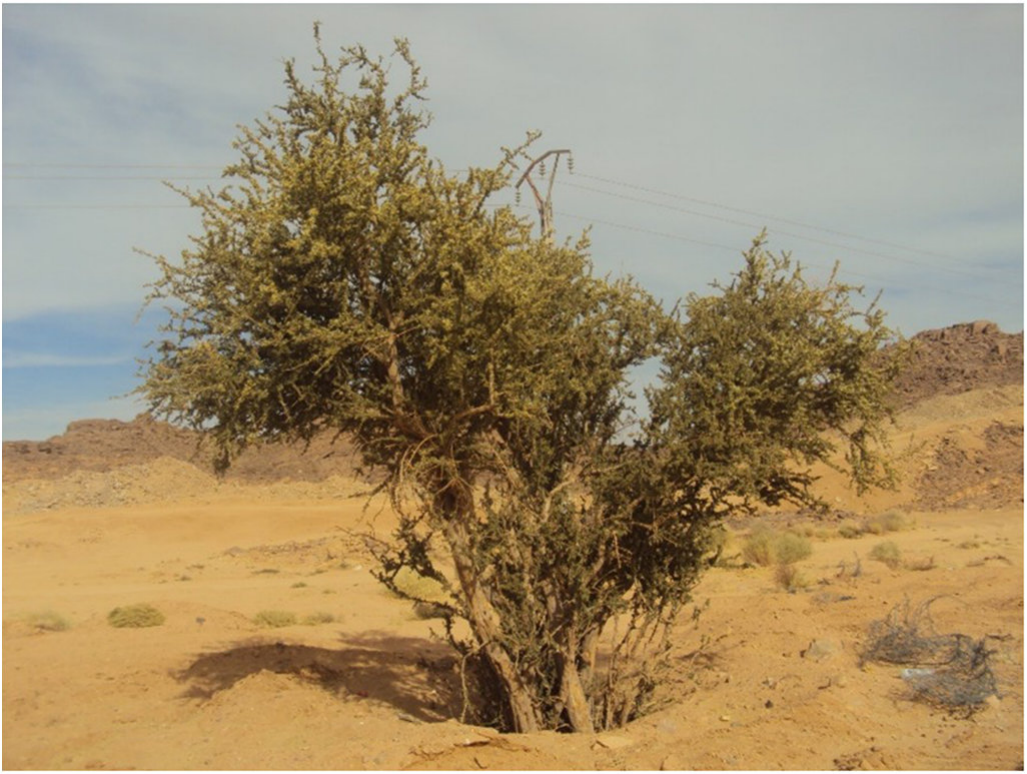
*Maerua crassifolia* Forssk. tree at the Tissenaouene station.

### 2.4 Laboratory Animals

Male and female Swiss albino mice (NMRI strain) weighing 19–‍26 g were used. Mice were obtained from the Pasteur Institute, Algiers, Algeria. They were accommodated in conventional enclosures and acclimatized for 2 weeks prior to experiments under controlled laboratory settings (12 h light/dark cycle, 20–24 °C, and 50%–65% relative humidity). After the acclimatization period, the mice were assigned at random to the designated control and treatment groups. This procedure was used to reduce the risk of selection bias and to promote balanced group composition. No blinding was applied during either treatment administration or the evaluation of outcomes. Throughout the experiment, the mice were provided with a standard pelleted diet and drinking water ad libitum.

### 2.5 Preparation of Infusion and Yield Estimation

To prepare the infusion as per the traditional method, *M. crassifolia* Forssk. gas chromatography–mass spectrometry (GC‐MS) aerial part powder was combined with boiling distilled water at 5% (w/v). The mixture was sealed and permitted to steep for 10 min. The infusion was then recovered and filtered through Whatman No. 3 filter paper before use in subsequent assays. In addition, to estimate infusion yield, the filtrate was subjected to evaporation after filtration, and what remained was dried to constant weight at 40 °C to collect the dry infusion. The infusion yield was estimated as a percentage of dry extract relative to the initial dry weight (d.w.) of the plant material.

### 2.6 Phytochemical Analysis

#### 2.6.1 Total Phenolic and Flavonoid Contents

The total phenolic content (TPC) and total flavonoid content (TFC) of the infusion were determined spectrophotometrically using the Folin–Ciocalteu [31] and aluminum chloride colorimetric methods, respectively [23]. For TPC, an aliquot of the appropriately diluted sample was reacted with a diluted Folin–Ciocalteu reagent, followed by a sodium carbonate solution, then incubated in the dark at room temperature before absorbance measurement at 765 nm. Gallic acid was used to prepare the calibration curve, and results were expressed as mg gallic acid equivalents (GAE) per 100 g of d.w. For TFC, the diluted sample was mixed with aluminum chloride solution, incubated at room temperature, and absorbance was read at 430 nm. Quercetin was used as the reference standard, and results were expressed as mg quercetin equivalents (QE) per 100 g of d.w.

#### 2.6.2 UPLC‐PDA‐MS/MS Analysis

Ultra‐performance liquid chromatography with photodiode array detection and tandem mass spectrometry (UPLC‐PDA‐MS/MS) were used to analyze the infusion. The analysis was performed using an ACQUITY UPLC system (Waters, Milford, MA, USA) equipped with a photodiode array detector (PDA) coupled to an Xevo TQ‐S tandem quadrupole mass spectrometer (Waters, UK) via electrospray ionization (ESI). An ACQUITY UPLC HSS T3 column (1.8 µm, 2.1 × 150 mm) was used at 45 °C with a 10 µL sample and a flow rate of 0.50 mL/min to separate the chemicals. Mobile phases consisted of A (H_2_O: acetonitrile (ACN) 95:5 + 0.1% acetic acid) and B (ACN + 0.1% acetic acid), using the gradient: 0–1.89 min (1% B), 1.89–17.84 min (30% B), 17.84–21.39 min (5% B), 21.39–21.56 min (1% B), and 21.56–25 min (1% B isocratic). The mass spectrometer was operated in both positive‐ and negative‐ion ESI modes, with capillary voltages of 3.0 kV and −2.5 kV, respectively. The source and desolvation temperatures were 150 °C and 500 °C, respectively. The cone gas (N_2_) and desolvation gas flow rates were 150 L/h and 800 L/h, respectively. Multiple reaction monitoring (MRM) transitions were optimized using standard injections, with collision‐induced dissociation via Ar (0.15 mL/min). Data acquisition used MassLynx 4.1 software. Finally, for the compound’s identification, retention times, UV–‍‍vis, and MS/MS spectra of sample peaks were compared with authenticated standards.

### 2.7 Antioxidant Efficacy

The antioxidant capacity of the infusion was assessed using several tests, namely, total antioxidant capacity (TAC), 2,2‐diphenyl‐1‐picrylhydrazyl (DPPH) radical scavenging, 2,2′‐azinobis(3‐ethylbenzothiazoline‐6‐sulfonic acid) (ABTS) radical scavenging, and the ferric‐reducing antioxidant power (FRAP) assays. TAC was determined by the phosphomolybdenum assay [32], in which the sample was incubated with a reagent containing sulfuric acid, sodium phosphate, and ammonium molybdate at high temperature (95 °C) before absorbance was measured at 695 nm. DPPH radical‐scavenging properties were assessed by mixing the sample with DPPH solution and measuring the absorbance at 517 nm after incubation in the dark [33]. ABTS radical‐scavenging activity was evaluated using a preformed ABTS radical cation solution adjusted to an absorbance of 0.70 at 734 nm. The solution was then mixed with the sample and absorbance was measured at the same wavelength [34]. FRAP was assessed using the ferric‐reducing assay, where the sample reacted with phosphate buffer and potassium ferricyanide, followed by the addition of trichloroacetic acid and ferric chloride. Absorbance was measured at 700 nm [23], and the results were expressed as milligrams of ascorbic acid equivalents per 100 g of d.w. (mg AAE/100 g d.w.) for TAC, DPPH, and FRAP, and as milligrams of trolox equivalents per 100 g of d.w. (mg TE/100 g d.w.) for ABTS.

### 2.8 Acute Oral Toxicity Test

The acute oral toxicity assessment was performed following OECD Test Guideline No. 423 [35]. A limit test was performed at 5000 mg/kg body weight (b.w.). Groups of three male and female Swiss albino mice (*n* = 3/group) received 0.5 mL of the prepared infusion of *M. crassifolia* Forssk. aerial parts by oral gavage after a 4 h period of fasting, while the control group received an equivalent volume of distilled water as the vehicle. The tests were repeated twice over 14 days each. Mice were closely observed for signs of toxicity or mortality during the first 4 h after administration of the infusion and afterward were accompanied by checks every day for 14 days. Clinical observations included changes in general appearance, fur, posture, activity, scratching, aggressiveness, sensitivity to noise or light, hypersalivation, respiration, heart rate, abdominal contractions, diarrhea, sleep, and coma. Body weight was recorded before treatment and on days 7 and 14 after administration of the infusion.

### 2.9 Analgesic Efficacy

To evaluate the analgesic action, two types of tests were chosen: the writhing test to assess peripheral analgesic action and the formalin test to assess both central and peripheral analgesic action.

#### 2.9.1 Writhing Test

The writhing assay was conducted using a standard experimental method [36]. Five groups of six female mice each received the following by oral gavage. The first group received 0.5 mL of 0.9% physiological saline and served as the control. The second group received 0.5 mL of paracetamol (100 mg/kg b.w.) as the reference group, and the last three groups received 0.5 mL of *M. crassifolia* Forssk. aerial parts infusion at doses of 250, 500, and 1000 mg/kg b.w., respectively. After 30 min, all mice received an intraperitoneal injection of 0.2 mL of 0.6% acetic acid to induce abdominal pain. The number of writhes was counted for 15 min, starting 5 min after injection, and the percentage of protection was determined from the mean number of writhes in the control group compared with that in the treated groups.

#### 2.9.2 Formalin Test

For the formalin test [21], the female mice were divided into five (6 mice/group) groups as follows: the first group received 0.5 mL of 0.9% physiological saline and served as the control. The second group received paracetamol as the reference drug (50 mg/kg b.w.) and the last three groups received *M. crassifolia* Forssk. infusion at doses of 250, 500, and 1000 mg/kg b.w., respectively. 1 h later, 20 µL of 2.5% formalin was administered subcutaneously into the dorsal surface of the right hind paw of each animal. Licking duration was documented during the initial phase (0–10 min) and the subsequent phase (15–30 min). The percentage protection was calculated based on the difference between the mean licking time in the control group and that observed in the treated groups.

### 2.10 Anti‐Inflammatory Efficacy

The anti‐inflammatory effect was estimated using the carrageenan‐induced paw model [21]. Five groups of six female mice each received the following oral treatments: group 1, 0.5 mL of 0.9% physiological saline (control); group 2, sodium diclofenac as the reference drug (50 mg/kg b.w.); groups 3–5, *M. crassifolia* Forssk. aerial parts infusion (250, 500, and 1000 mg/kg b.w.). After 30 min, 0.1 mL of 1% (in 0.9% saline) freshly prepared suspension of carrageenan was injected subplantarly into the right hind paw of mice in all groups to induce inflammation and paw edema. The left hind paw remained untreated and served as the internal control for assessing changes in paw thickness. After 4 h, experimental mice were euthanized by cervical dislocation, and both hind paws were severed at the ankle joint (talocrural articulation) and weighed.

The percentage paw‐weight increase (PE) was calculated using the following equation:

$$\begin{array}{l} \mathrm{PE}=\frac{\mathrm{RPW}-\mathrm{LPW}}{\mathrm{LPW}}\times 100 \end{array}$$

where RPW is the weight of the carrageenan‐injected right paw, and LPW is the weight of the untreated left paw.

The percentage inhibition of edema (PIE) was calculated using the equation below:

$$\begin{array}{l} \mathrm{PIE}=\frac{{\mathrm{PE}}_{c}-{\mathrm{PE}}_{t}}{{\mathrm{PE}}_{c}}\times 100 \end{array}$$

where PE_c_ is the mean PE of the control group; PE_t_ is the mean PE of the treated groups (reference or infusion).

### 2.11 Statistical Analysis

Data are presented as means ± standard deviation (SD) for in vitro experiments and as mean values ± Standard Error of the Mean (SEM) for in vivo investigations using GraphPad Prism 7 software. Differences between the infusion‐treated groups and the control group were assessed using one‐way ANOVA, followed by pairwise *t*‐test. In addition, one‐way ANOVA followed by Tukey’s post hoc test was used for multiple‐group comparisons. A *p*‐value of < 0.05 was deemed statistically significant.

## 3 Results and Discussion

### 3.1 Phytochemical Study

In the present study, the yield of *M. crassifolia* Forssk. aerial part infusion was first determined, followed by an assessment of its TPC and TFC (Table 1). The infusion of the aerial parts collected in the Tamanrasset region yielded 11.93% ± 1.08% weight by weight (w/w), exceeding the yields reported for the decoction of *M. crassifolia* leaves from Niger (7%) [37] and for the methanolic extract of the aerial parts from Saudi Arabia (4.43%) [26]. The TPC was 620.51 ± 2.54 mg GAE/100 g d.w., indicating a high phenolic content. This value was higher than those reported in previous studies, suggesting that the infusion prepared in the present study is particularly rich in phenolic constituents [26, 37, 38, 39]. The TFC was 87.11 ± 0.76 mg QE/100 g d.w., which also exceeded previously reported values for similar extracts from different localities [37, 38, 39].

**TABLE 1:** Yield, total phenolic, and flavonoid contents of *Maerua crassifolia* Forssk. aerial parts infusion.

Sample	Yield %, w/w	Total polyphenol content, mg GAE/100 g d.w.	Total flavonoid content, mg QE/100 g d.w.
*M. crassifolia* Forssk. infusion	11.93 ± 1.08	620.51 ± 2.54	87.11 ± 0.76

*Note:* Values are presented as mean ± SD of three replicates. mg GAE/100 g d.w., mg gallic acid equivalents per 100 g dry weight; mg QE/100 g d.w., mg quercetin equivalents per 100 g of dry weight.

Abbreviation: w/w, weight by weight.

The phenolics identified in the infusion of aerial parts of *M. crassifolia* Forssk. (Figure 2), together with their retention times, are presented in Table 2. The UPLC‐PDA‐MS/MS analysis of the infusion enabled, for the first time, the identification of 19 phenolic compounds, including the flavonoid glycosides rutin, hyperoside and quercetin‐3‐*O*‐glucopyranoside. The phenolic‐acid fraction comprised caffeic, ellagic, vanillic, protocatechuic, gallic, chlorogenic, syringic, *p*‐coumaric, ferulic, and salicylic acids. In addition, quercetin, phloretin, epigallocatechin gallate, kaempferol, Delphinidin‐3‐*O*‐rutinoside chloride and theaflavin digallate were detected. Overall, these results indicate that the infusion is characterized by a predominance of flavonoid glycosides, accompanied by a diverse profile of phenolic acids and other polyphenolic constituents.

**FIGURE 2:**
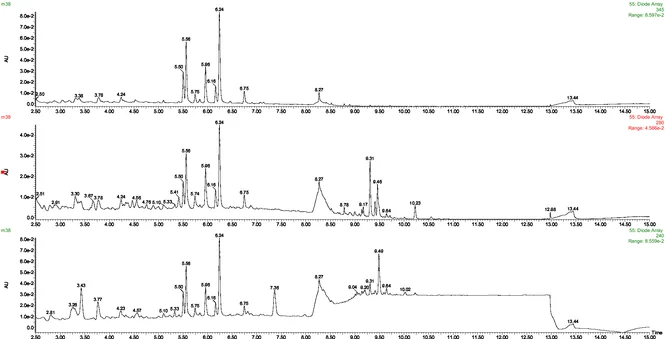
UPLC‐PDA epigallocatechin gallate chromatographic profiles of *Maerua crassifolia* Forssk. aerial parts infusion.

**TABLE 2:** Phenolics identified in the infusion of *Maerua crassifolia* Forssk. aerial parts.

N°	Compound	Retention time, min
1	Chlorogenic acid	3.9
2	Protocatechuic acid	4.72
3	–	4.95
4	Salicylic acid	4.97
5	Vanillic acid	5.06
6	Syringic acid	5.31
7	Kaempferol	5.51
8	*p*‐Coumaric acid	6.05
9	Delphinidin‐3‐*O*‐rutinoside chloride	6.12
10	Hyperoside	6.18
11	Ellagic acid	6.36
12	Quercetin‐3‐*O*‐glucopyranoside	6.57
13	Ferulic acid	6.7
14	Caffeic acid	6.76
15	Rutin	6.85
16	Gallic acid	8.42
17	Quercetin	8.43
18	Theaflavin digallate	8.50
19	Phloretin	8.55

Based on a literature search, the phytochemical profile of *M. crassifolia* Forssk. aerial parts has been studied here for the first time using UPLC‐PDA‐MS/MS analysis; therefore, a direct comparison with previous investigations on the same species is not possible. The available literature on *M. crassifolia* remains very limited. Only one detailed phytochemical study has been reported, in which *M. crassifolia* collected in Egypt was investigated and a new ionol glucoside was isolated using classical fractionation and spectroscopic techniques [40]. Other studies focused on phytochemical screening of different extracts and reported the presence of polyphenols, flavonoids, coumarins, saponins, sterols, tannins, triterpenes, and alkaloids [37, 41, 42].

Several other *Maerua* species have been more extensively studied for their phytochemical composition using GC–MS. For example, GC–MS has been used to investigate the chemical profile of *M. angolensis* methanolic extract from Nigeria as well as the profiles of *M. oblongifolia* extracts prepared using ethanol, acetone, ethyl acetate, and dichloromethane [42, 43]. Overall, the present phytochemical analysis demonstrates that the infusion of *M. crassifolia* Forssk. from Tamanrasset is rich in phenolic compounds, particularly flavonol glycosides such as rutin, quercetin derivatives, hyperoside, and delphinidin‐3‐*O*‐rutinoside chloride, along with a diverse array of phenolic acids.

### 3.2 Antioxidant Activity

The antioxidant efficacy of *M. crassifolia* Forssk. aerial parts infusion was tested using different assays, with all results shown in Table 3. The TAC value was the highest (8920.72 ± 210.90 mg AAE/100 g d.w.), indicating a strong overall reducing capacity of the infusion in the phosphomolybdenum system (*R* ^2^ = 0.9997). The infusion also demonstrated considerable free‐radical scavenging efficacy, with DPPH and ABTS values of 517.64 ± 3.68 mg AAE/100 g d.w. (*R* ^2^ = 0.9997) and 599.70 ± 2.20 mg TE/100 g d.w. (*R* ^2^ = 0.9966), respectively. The FRAP value was 977.62 ± 2.93 mg AAE/100 g d.w. (*R* ^2^ = 0.9981), reflecting a strong electron‐donating ability of the infusion constituents.

**TABLE 3:** Antioxidant activity of *Maerua crassifolia* Forssk. aerial parts infusion.

Sample	TAC, mg AAE/100 g d.w.	DPPH, mg AAE/100 g d.w.	ABTS, mg TE/100 g d.w.	FRAP, mg AAE/100 g d.w.
*M. crassifolia* Forssk. infusion	8920.72 ± 210.90	517.64 ± 3.68	599.70 ± 2.20	977.62 ± 2.93

*Note:* Values are presented as mean ± SD of three replicates. ABTS, 2,2′‐azino‐bis(3‐ethylbenzothiazoline‐6‐sulfonic acid) radical‐scavenging activity; DPPH, 2,2‐diphenyl‐1‐picrylhydrazyl radical‐scavenging activity; mg AAE/100 g d.w., milligrams of ascorbic acid equivalents per 100 g of dry weight; mg TE/100 g d.w., milligrams of trolox equivalents per 100 g of dry weight.

Abbreviations: FRAP, ferric reducing antioxidant power; TAC, total antioxidant capacity.

The strong antioxidant activity observed in all assays is likely linked to this phenolic richness. The high total antioxidant capacity, together with the DPPH, ABTS, and FRAP results, suggests that the infusion can act through multiple mechanisms, including hydrogen donation, electron transfer, and overall reducing power. This antioxidant performance of *M. crassifolia* Forssk. is consistent with previous reports on this species using different solvents and plant parts [37, 44]. The methanol root extract showed moderate radical‐scavenging activity against DPPH, while the leaf decoction also exhibited good antioxidant properties against DPPH and ABTS radicals [37, 44]. Numerous studies have demonstrated that the antioxidant efficacy of plants is largely attributed to polyphenols, flavonoids, and tannins, and a strong positive correlation has been reported between polyphenol content and antioxidant potential [20, 23, 37]. The present results are likely linked to the occurrence of phenolic acids such as chlorogenic, ellagic, caffeic, vanillic, and syringic acids [45, 46, 47, 48, 49, 50], as well as flavonoids such as rutin, apigenin, luteolin, quercetin, and their derivatives [51, 52, 53]. These chemicals are recognized for contributing to radical‐scavenging and reducing activities.

### 3.3 Toxicity

The acute oral toxicity experiment revealed that the infusion of the studied plant exhibited no indications of harm or mortality in the treated mice throughout the experimental period at the tested dose. No abnormal behavioral changes, like tremors, diarrhea, or loss of appetite, were noted following administration of the infusion. In addition, no significant alterations in physical appearance or general activity were observed, suggesting that the infusion was well tolerated. These results suggest that the examined infusion possesses a favorable safety profile and may be considered nontoxic and safe at a dose of 5000 mg/kg b.‍w., with the median lethal dose (LD_50_) estimated to be greater than 5000 mg/kg b.w. This suggests a wide margin of safety and supports its potential use in traditional or future therapeutic applications. Similar safety observations have been reported for other extracts of *M. crassifolia* and related species, reinforcing the idea that members of this genus may be well tolerated when properly prepared and administered. Comparable results were observed after administration of methanolic and ethanolic leaf extracts of the same plant from Nigeria, with no toxicity detected at doses up to 5000 mg/kg b.w [41, 54]. Likewise, root extracts of *M. triphylla* from Kenya were also reported to be nontoxic at LD_50_ > 2000 mg/kg b.w [55]. As shown in Figure 3, the administration of the infusion at 5000 mg/kg b.w. did not result in any apparent adverse effects on body weight during the 14‐day observation period. Body weight increased progressively in both male and female mice in the treated and control groups. Statistical analysis revealed no significant differences in body weight gain between the treated and control groups (*p* > 0.05) (Table 4).

**FIGURE 3:**
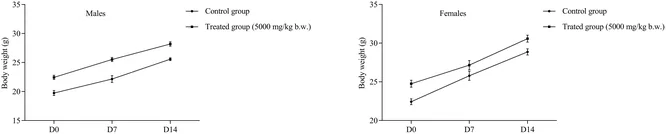
Body weight of mice treated orally with *Maerua crassifolia* Forssk. aerial parts infusion. Values are expressed as mean ± SEM, *n* = 6 mice/group.

**TABLE 4:** Body weight gain (g) of mice treated orally with *Maerua crassifolia* Forssk. aerial parts infusion.

Parameter	Groups	Control group	Treated group, 5000 mg/kg b.w.
Body weight gain (g)	Male mice group	5.85 ± 0.42	5.57 ± 0.17^ns^
	Female mice group	6.43 ± 0.70	5.83 ± 0.49^ns^

*Note:* Values are expressed as mean ± SEM, *n* = 6 mice/group.

ns No significant difference, compared with control group (*p* > 0.05, *t*‐test).

### 3.4 Analgesic Activity

The analgesic efficacy of the plant infusion was assessed by the acetic acid‐induced writhing test, and the findings are presented in Figure 4. Administration of the infusion at doses of 250 and 500 mg/kg b.w. reduced the number of induced abdominal writhes, with inhibition percentages of 61.88% ± 1.90% and 69.82% ± 3.05%, respectively. The best analgesic activity was observed at 1000 mg/kg b.w. (74.02% ± 2.17%), which is higher than that of paracetamol (100 mg/kg b.w.), whose inhibition percentage is 68.24% ± 3.06% (*p* = 0.005) (Table S1). The results obtained from this study on analgesic activity were consistent with those reported for the methanolic leaf extract of *M. crassifolia* from Nigeria, which produced 80% inhibition at 400 mg/kg b.w [54]. Likewise, both aqueous and methanolic root extracts of *M. triphylla* from Kenya markedly reduced acetic acid‐induced writhing in mice [55]. In fact, at 500 mg/kg b.w., the aqueous root extract showed 95.97% ± 1.16% inhibition, while the methanolic root extract showed 92.55% ± 1.20% inhibition. These findings suggest that the infusion may suppress prostaglandin production, a major mediator of inflammatory pain, possibly due to its content of bioactive compounds such as flavonoids and phenolic compounds [55, 56]. The analgesic effect may also be related to the presence of chlorogenic acid, kaempferol, quercetin, and rutin [52, 57, 58, 59]. Overall, these results support previous reports describing similar analgesic activities in other plant species [60, 61, 62].

**FIGURE 4:**
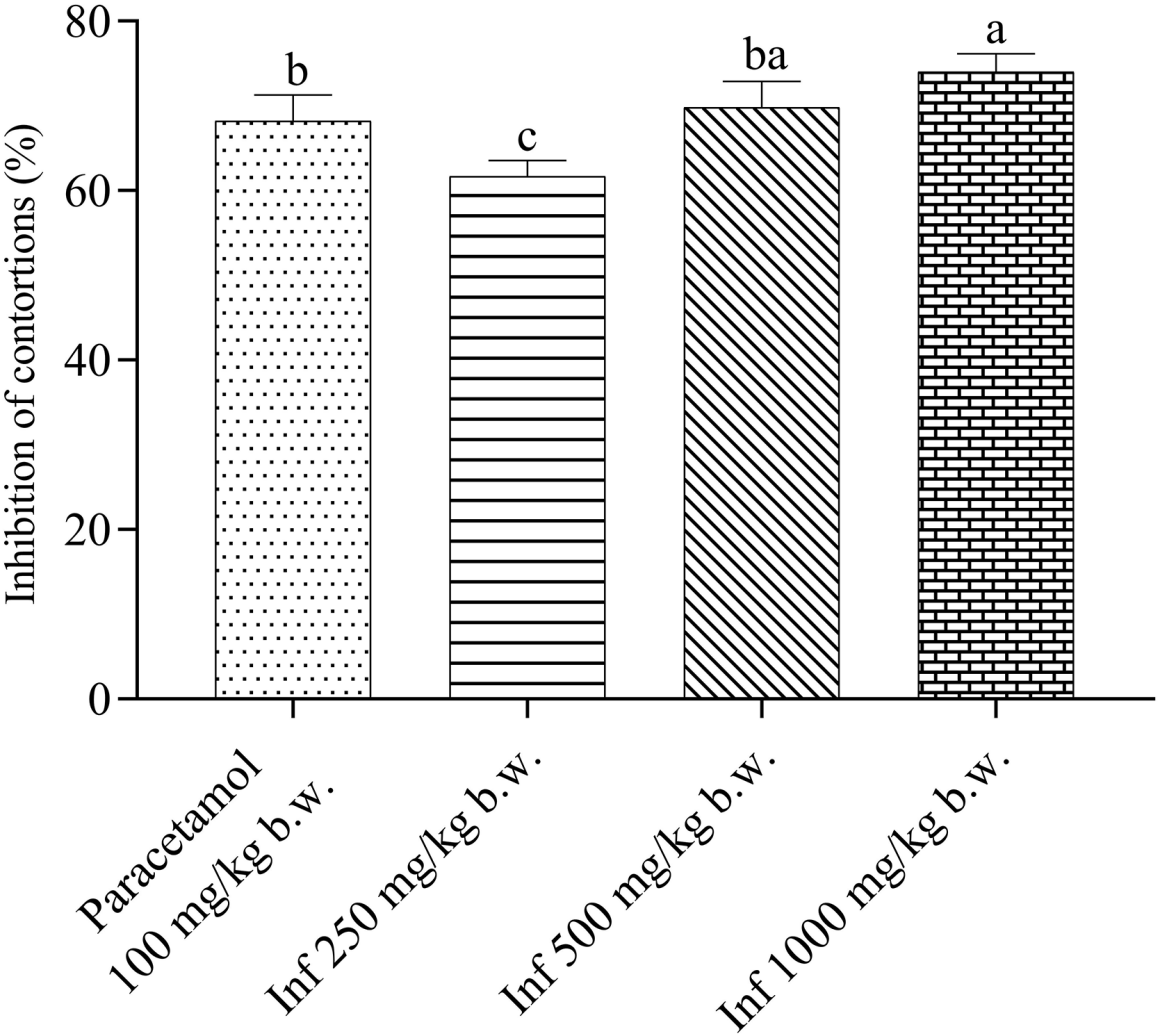
Analgesic activity of *Maerua crassifolia* Forssk. aerial parts infusion and paracetamol on the acetic acid‐induced writhing test. Values are expressed as mean ± SEM, *n* = 6 mice/group. Distinct letters within the same columns signify a statistically significant difference (*p* < 0.05, Tukey’s test).

The analgesic effect of the infusion was also evaluated using the formalin‐induced paw licking test, which allows differentiation between neurogenic pain (early phase, 0–10 min) and inflammatory pain (late phase, 15–30 min) (Table 5). The infusion of *M. crassifolia* aerial parts exhibited a dose‐dependent antinociceptive action. In the early phase, at 250 and 500 mg/kg b.w., the licking time decreased to 58.00 ± 0.83 s and 36.20 ± 1.59 s, with an inhibition of 31.44% ± 0.99% and 57.21% ± 1.36%, respectively. The highest dose produced the strongest effect, reducing the licking time to 18.80 ± 0.58 s with an inhibition rate of 77.78% ± 0.70%, as compared with paracetamol (63.36% ± 1.18%) (*p* < 0.001) (Table S2). Similarly, *M. crassifolia* aerial parts infusion demonstrated a substantial inhibitory impact during the inflammatory phase at 250 and 500 mg/kg b.w., the licking time was reduced to 31.20 ± 1.15 s and 26.00 ± 1.09 s, corresponding to an inhibition of 52.73% ± 1.75% and 60.61% ± 1.66%, respectively. The highest dose demonstrated the greatest efficacy, with a licking time of 17.60 ± 0.75 s and an inhibition percentage of 73.33% ± 1.13%, compared with paracetamol (63.94% ± 1.45%) (*p* < 0.001) (Table S3).

**TABLE 5:** Anti‐nociceptive activity of *Maerua crassifolia* Forssk. aerial parts infusion, negative control, and paracetamol in the formalin‐induced licking assay.

Groups	Dose, mg/kgb.w.	Early phase, 0–10 min		Late phase, 15–30 min	
		Licking/biting time, s	Inhibition, %	Licking/biting time, s	Inhibition, %
Negative control	0	84.60 ± 1.43^e^	—	66.00 ± 1.30^d^	—
Paracetamol	50	31.00 ± 1.00^b^	63.36 ± 1.18^b^	23.80 ± 0.97^b^	63.94 ± 1.45^b^
*M. crassifolia* Forssk. infusion	250	58.00 ± 0.83^d^	31.44 ± 0.99^d^	31.20 ± 1.15^c^	52.73 ± 1.75^c^
	500	36.20 ± 1.59^c^	57.21 ± 1.36^c^	26.00 ± 1.09^b^	60.61 ± 1.66^b^
	1000	18.80 ± 0.58^a^	77.78 ± 0.70^a^	17.60 ± 0.75^a^	73.33 ± 1.13^a^

*Note:* Values are expressed as mean ± SEM, *n* = 6 mice/group. Distinct letters within the same columns signify a statistically significant difference (*p* < 0.05, Tukey’s test).

The antinociceptive activity of different extracts of *Maerua angolensis* from Nigeria was investigated at various doses, and a reduction in licking time was observed in both the neurogenic and inflammatory phases [63]. The early phase of the formalin test is mainly associated with direct activation of nociceptors and reflects neurogenic pain, whereas the late phase is linked to inflammatory processes mediated by prostaglandins, histamine, serotonin, and other inflammatory mediators [64]. The significant reduction in both phases suggests that the extracts or infusions may interfere with nociceptive transmission as well as the synthesis of inflammatory mediators. This anti‐nociceptive effect may be attributed to bioactive secondary metabolites such as chlorogenic acid, kaempferol, quercetin, and rutin [52, 57, 58, 59]. These compounds are known to exert analgesic effects through several pathways, including inhibition of cyclooxygenase‐1 (COX‐‍‍1) and COX‐2, suppression of prostaglandin production, and modulation of oxidative stress, which plays an important role in nociception [55, 56, 57, 58, 59, 60, 61, 62, 63, 64, 65].

The analgesic activity observed in both the acetic acid writhing test and the formalin assay confirms that the infusion exerts peripheral and central antinociceptive effects. The reduction in writhing suggests inhibition of prostaglandin‐mediated pain, while the suppression of both the early and late phases in the formalin test indicates interference with neurogenic and inflammatory pain pathways. This broad analgesic profile may again be explained by the presence of flavonoids and phenolic acids, which are known to modulate nociceptive signaling, inflammatory mediator release, and oxidative stress [56]. The results of this study are noteworthy and provide scientific support for the traditional use of the plant to relieve pain.

### 3.5 Anti‐Inflammatory Efficacy

The anti‐inflammatory potential of the tested infusion was evaluated by measuring the percentage of paw edema and the percentage of edema inhibition (Table 6). The plant infusion demonstrated a dose‐dependent anti‐inflammatory influence. At 250 mg/kg b.w., the edema percentage decreased to 30.25% ± 1.08%, with an inhibition rate of 31.24% ± 2.47%, indicating a moderate anti‐inflammatory activity relative to the control group (*p* < 0.001). Increasing the dose to 500 mg/kg b.w. further reduced the edema percentage to 24.45% ± 1.01%, with an inhibition of 44.42% ± 2.30%, which is comparable to the effect of sodium diclofenac with an inhibition of 49.08% ± 2.01% (*p* = 0.427). The maximum dosage of 1000 mg/kg b.w. produced the most pronounced anti‐inflammatory effect, with an edema percentage of 19.26% ± 1.50% and an inhibition rate of 56.22% ± 1.52%, which is close to the inhibitory effect observed with sodium diclofenac (49.08% ± 2.01%) (*p* = 0.119) (Tables S4, S5, and S6).

**TABLE 6:** Paw edema increases and the inhibition of paw edema of *Maerua crassifolia* Forssk. aerial parts infusion, negative control, and sodium diclofenac against carrageenan‐induced edema.

Sample	Dose, mg/kg b.w.	Paw edema increase (PE, %)	Inhibition of paw edema (PIE, %)
Negative control	0	43.99 ± 1.84^d^	—
Sodium diclofenac	50	22.40 ± 1.98^ab^	49.08 ± 2.01^bc^
*M. crassifolia* Forssk. infusion	250	30.25 ± 1.08^c^	31.24 ± 2.47^d^
	500	24.45 ± 1.01^b^	44.42 ± 2.30^c^
	1000	19.26 ± 1.50^a^	56.22 ± 1.52^a^

*Note:* Values are expressed as mean ± SEM, *n* = 6 mice/group. Distinct letters within the same columns signify a statistically significant difference (*p* < 0.05, Tukey’s test).

In the carrageenan‐induced edema model, the infusion produced a clear dose‐dependent anti‐inflammatory effect. The anti‐inflammatory activity observed for the studied plant was comparable to that reported in similar studies, while other *Maerua* species have shown variable levels of inhibition in the carrageenan model [54, 66]. For instance, the results of the ethanolic extract of Indian *Maerua apetala* at a dose of 300 mg/kg b.w., using the carrageenan‐induced rat paw edema model, showed a higher anti‐inflammatory activity (84.17%). Furthermore, the anti‐inflammatory effects observed in this study were greater than those reported for the aqueous and methanolic extracts of *M. triphylla*, which showed lower percentages of inhibition [55]. Inflammation is mediated by compounds such as histamine, prostaglandins, reactive oxygen species, and pro‐inflammatory cytokines, all of which contribute to edema, pain, and vascular changes [67]. Phenolic compounds, especially flavonoids, may reduce inflammation largely through their antioxidant activity by scavenging free radicals and decreasing oxidative stress [56, 68]. In particular, hyperoside, kaempferol, and quercetin are widely recognized for their anti‐inflammatory properties, mainly through inhibition of cyclooxygenase and lipoxygenase enzymes, reduction of pro‐inflammatory cytokines, and neutralization of free radicals [51, 52, 68, 69].

## 4 Conclusion

The present study provides an integrated evaluation of the aerial parts infusion of *M. crassifolia* Forssk. from Algeria, combining phytochemical characterization with antioxidant, anti‐inflammatory, analgesic, and acute oral toxicity assessments to investigate its biological properties and safety. Phytochemical analysis revealed the presence of phenolic and flavonoid compounds that may contribute to the in vitro antioxidant and in vivo anti‐inflammatory and analgesic effects observed in the experimental models. The infusion also exhibited a favorable safety profile under the experimental conditions, with no signs of acute oral toxicity at the tested dose. Overall, these findings provide experimental support for the traditional use of *M. crassifolia* Forssk. and highlight its potential as a source of bioactive compounds. Further investigations are needed to identify the compounds responsible for the observed activities and clarify their mechanisms of action.

## Funding

This work was supported by both the Ministry of Higher Education and Scientific Research of Algeria and the University of Sciences and Technology Houari Boumediene, Algiers (Algeria), for supporting project N F00220130059.

## Conflicts of Interest

The authors declare no conflicts of interest.

## Supporting information

Supplementary Material

## Data Availability

The data that support the findings of this study are available from the corresponding author upon reasonable request.
