# Supplementary material for: Phytochemical Profiling and Safety Assessment of Maerua crassifolia Forssk. Aerial Parts Infusion With Pharmacological Validation of Its Analgesic and Anti‐Inflammatory Activities
Source: ChemistryOpen. 2026 Oct 26;15(11):e70326. doi: 10.1002/open.70326 (PMC13700108; doi:10.1002/open.70326)
Supplement: Supplementary file 1 [file OPEN-15-e70326-s001.pdf]

Phytochemical Profiling and Safety Assessment of *Maerua crassifolia* Forssk. Aerial Parts Infusion with Pharmacological Validation of its Analgesic and Anti-inflammatory Activities

Leila Benazzi[1] | Saida Ouafi[1] | Redouane Semaoui[1] | Faiza Baali[2] | Jordi Eras[3,4] | Ramon Canela-Garayoa[3] | Nadjette Djemouai[2,5*]

[1]Laboratory of Research on Arid Zones (LRZA), Faculty of Biological Sciences, University of Science and Technology Houari Boumediene (USTHB), BP 32 El-Alia, 16111 Bab Ezzouar, Algiers, Algeria | [2]Department of Biology, Faculty of Natural and Life Sciences and Earth Sciences, University of Ghardaia, BP 455, Ghardaïa 47000, Algeria | [3]Department of Chemistry, DBA center, University of Lleida, Alcalde RoviraRoure 191, 25198 Lleida, Spain| [4]Scientific Technical Services TCEM, University of Lleida, Alcalde Rovira Roure 191, 25198 Lleida, Spain | [5]Laboratory of Microbial Systems Biology (LBSM), Higher National School Cheikh Mohamed El Bachir El Ibrahimi, BP 92, Kouba, Algiers, Algeria.

Correspondence: Nadjette Djemouai (djemouai.nadjette@univ-ghardaia.dz)

Supplementary Tables

Supplementary Table 1. Detailed statistical evaluation of analgesic effects in the acetic acid–induced writhing assay.

**Comparison**	**Mean difference**	**95% Confidence interval**	***p*-value**	**Significance**
Paracetamol 100 mg/kg b.w. vs. Inf 250 mg/kg b.w.	6.562	2.361 to 10.76	0.002	Yes
Paracetamol 100 mg/kg b.w. vs. Inf 500 mg/kg b.w.	-1.575	-5.776 to 2.626	0.723	No
Paracetamol 100 mg/kg b.w. vs. Inf 1000 mg/kg b.w.	-5.774	-9.975 to -1.573	0.005	Yes
Inf 250 mg/kg b.w. vs. Inf 500 mg/kg b.w.	-8.136	-12.34 to -3.936	<0.001	Yes
Inf 250 mg/kg b.w. vs. Inf 1000 mg/kg b.w.	-12.34	-16.54 to -8.135	<0.001	Yes
Inf 500 mg/kg b.w. vs. Inf 1000 mg/kg b.w.	-4.199	-8.4 to 0.001421	0.050	No

Inf: infusion, b.w.: body weight.

Supplementary Table 2. Detailed statistical evaluation of analgesic effects in the early phase of the formalin assay.

**Comparison**	**Mean difference**	**95% Confidence interval**	***p*-value**	**Significance**
Paracetamol 50 mg/kg b.w. vs. Inf 250 mg/kg b.w.	31.91	27.52 to 36.31	<0.001	Yes
Paracetamol 50 mg/kg b.w. vs. Inf 500 mg/kg b.w.	6.147	1.75 to 10.54	0.005	Yes
Paracetamol 50 mg/kg b.w. vs. Inf 1000 mg/kg b.w.	-14.42	-18.82 to -10.02	<0.001	Yes
Inf 250 mg/kg b.w. vs. Inf 500 mg/kg b.w.	-25.77	-30.16 to -21.37	<0.001	Yes
Inf 250 mg/kg b.w. vs. Inf 1000 mg/kg b.w.	-46.34	-50.73 to -41.94	<0.001	Yes
Inf 500 mg/kg b.w. vs. Inf 1000 mg/kg b.w.	-20.57	-24.96 to -16.17	<0.001	Yes

Inf: infusion, b.w.: body weight.

Supplementary Table 3. Detailed statistical evaluation of analgesic effects in the late phase of the formalin assay.

**Comparison**	**Mean difference**	**95% Confidence interval**	***p*-value**	**Significance**
Paracetamol 50 mg/kg b.w. vs. Inf 250 mg/kg b.w.	11.21	5.051 to 17.37	<0.001	Yes
Paracetamol 50 mg/kg b.w. vs. Inf 500 mg/kg b.w.	3.333	-2.828 to 9.494	0.434	No
Paracetamol 50 mg/kg b.w. vs. Inf 1000 mg/kg b.w.	-9.394	-15.55 to -3.233	<0.001	Yes
Inf 250 mg/kg b.w. vs. Inf 500 mg/kg b.w.	-7.879	-14.04 to -1.718	0.010	Yes
Inf 250 mg/kg b.w. vs. Inf 1000 mg/kg b.w.	-20.61	-26.77 to -14.45	<0.001	Yes
Inf 500 mg/kg b.w. vs. Inf 1000 mg/kg b.w.	-12.73	-18.89 to -6.566	<0.001	Yes

Inf: infusion, b.w.: body weight.

Supplementary Table 4. Raw paw-weight data from the carrageenan-induced paw oedema assay for in vivo anti-inflammatory activity.

Groups	Right paw weight (g)						Left paw weight (g)					
Control group	0.152	0.169	0.17	0.163	0.151	0.161	0.106	0.112	0.119	0.114	0.108	0.111
Sodium diclofenac 50 mg/kg b.w.	0.143	0.145	0.142	0.138	0.143	0.142	0.115	0.118	0.115	0.113	0.12	0.116
Inf 250 mg/kg b.w.	0.139	0.135	0.137	0.138	0.136	0.137	0.105	0.107	0.104	0.106	0.104	0.105
Inf 500 mg/kg b.w.	0.142	0.14	0.142	0.143	0.141	0.141	0.112	0.115	0.114	0.113	0.115	0.113
Inf 1000 mg/kg b.w.	0.132	0.127	0.132	0.129	0.136	0.131	0.110	0.108	0.11	0.109	0.112	0.110

Inf: infusion, b.w.: body weight.

Supplementary Table 5. Detailed statistical evaluation of paw edema (PE, %) in the carrageenan-induced paw oedema assay.

Comparison	Mean difference	95% Confidence interval	*p*-value	Significance
Negative control vs. sodium diclofenac 50 mg/kg mg/kg b.w.	21.59	16.64 to 26.54	<0.001	Yes
Negative control vs. Inf 250 mg/kg b.w.	13.74	8.793 to 18.69	<0.001	Yes
Negative control vs. Inf 500 mg/kg b.w.	19.54	14.59 to 24.49	<0.001	Yes
Negative control vs. Inf 1000 mg/kg b.w.	24.73	19.78 to 29.68	<0.001	Yes
Sodium diclofenac 50 mg/kg mg/kg b.w. vs. Inf 250 mg/kg b.w.	-7.848	-12.8 to -2.9	0.001	Yes
Sodium diclofenac 50 mg/kg mg/kg b.w. vs. Inf 500 mg/kg b.w.	-2.049	-6.997 to 2.899	0.729	No
Sodium diclofenac 50 mg/kg mg/kg b.w. vs. Inf 1000 mg/kg b.w.	3.142	-1.806 to 8.09	0.349	No
Inf 250 mg/kg b.w. vs. Inf 500 mg/kg b.w.	5.799	0.8509 to 10.75	0.017	Yes
Inf 250 mg/kg b.w. vs. Inf 1000 mg/kg b.w.	10.99	6.042 to 15.94	<0.001	Yes
Inf 500 mg/kg b.w. vs. Inf 1000 mg/kg b.w.	5.191	0.243 to 10.14	0.037	Yes

Inf: infusion, b.w.: body weight.

Supplementary Table 6. Detailed statistical evaluation of percentage edema inhibition (PEI, %) in the carrageenan-induced paw oedema assay**.**

**Comparison**	**Mean difference**	**95% Confidence interval**	***p*-value**	**Significance**
Sodium diclofenac 50 mg/kg b.w. vs. Inf 250 mg/kg b.w.	17.84	9.3 to 26.38	0.001	Yes
Sodium diclofenac 50 mg/kg b.w. vs. Inf 500 mg/kg b.w.	4.658	-3.883 to 13.2	0.427	No
Sodium diclofenac 50 mg/kg b.w. vs. Inf 1000 mg/kg b.w.	-7.142	-15.68 to 1.398	0.119	No
Inf 250 mg/kg b.w. vs. Inf 500 mg/kg b.w.	-13.18	-21.72 to -4.642	0.002	Yes
Inf 250 mg/kg b.w. vs. Inf 1000 mg/kg b.w.	-24.98	-33.52 to -16.44	<0.001	Yes
Inf 500 mg/kg b.w. vs. Inf 1000 mg/kg b.w.	-11.8	-20.34 to -3.26	0.006	Yes

Inf: infusion, b.w.: body weight.
